# Brief Review: Racial Disparities in the Presentation and Outcomes of Patients with Thoracic Aortic Aneurysms

**DOI:** 10.3390/jcdd12040140

**Published:** 2025-04-07

**Authors:** Nora Bacour, Rutger T. Theijsse, Simran Grewal, Robert J. M. Klautz, Nimrat Grewal

**Affiliations:** 1 Department of Cardiothoracic Surgery, Amsterdam University Medical Center Location AMC, 1105 AZ Amsterdam, The Netherlands; n.bacour@amsterdamumc.nl (N.B.); r.t.theijse@amsterdamumc.nl (R.T.T.); r.klautz@amsterdamumc.nl (R.J.M.K.); 2 Department of Orthopaedic Surgery, Onze Lieve Vrouwe Gasthuis, 1091 AC Amsterdam, The Netherlands; s.k.grewal@olvg.nl; 3 Department of Cardiothoracic Surgery, Leiden University Medical Center, 2333 ZG Leiden, The Netherlands; 4 Department of Anatomy and Embryology, Leiden University Medical Center, 2333 ZG Leiden, The Netherlands

**Keywords:** thoracic aortic aneurysms, race, aortopathy

## Abstract

(1) Background: Thoracic aortic aneurysms (TAAs) pose critical health risks and are often asymptomatic until a rupture or dissection occurs. Guidelines recommend surgical repair based on specific aortic diameters and risk factors, emphasizing the importance of early detection and intervention. Despite established clinical risk factors for the early detection of TAAs, the influence of racial disparities on TAAs remains underexplored. This study aims to provide a comprehensive summary of existing research on racial disparities in the presentation and outcomes of TAAs. (2) Methods: This literature review was conducted using a systematic search strategy to explore racial differences in the presentation and surgical outcomes of patients with TAAs. (3) Results: The findings demonstrated that black patients were younger at presentation and had a higher incidence of ruptured TAAs than non-black patients. Furthermore, compared to non-black patients, black patients had higher rates of cardiac arrhythmia and COPD, as well as comorbidities such as diabetes, hypertension, and renal insufficiency. For black patients undergoing open surgery, the surgical results showed improved 5-year survival rates after repair but higher perioperative mortality rates. All-cause or in-hospital mortality did not significantly differ between the racial groups, according to four studies. (4) Discussion: This review highlights significant racial disparities in TAA presentation and outcomes, underscoring the need for personalized risk stratification models. Standardized racial and ethnic definitions are essential for consistent and reliable research. Future studies should focus on identifying the underlying mechanisms driving racial disparities and on refining risk assessment models to enhance diagnostic and therapeutic strategies, ultimately improving patient outcomes across diverse populations.

## 1. Introduction

Thoracic aortic diseases encompass a wide spectrum of conditions, with thoracic aortic aneurysms (TAAs) and dissections being the most critical [1]. TAA occurs when the thoracic aorta, including the ascending aorta, aortic root, or aortic arch, dilates progressively [2]. TAAs often remain asymptomatic until a catastrophic event such as a dissection or rupture occurs, with mortality rates exceeding 90% without intervention [2]. Hence, early detection, close monitoring, and appropriate (surgical) intervention are imperative. Current American and European aortic guidelines recommend surgical repair of the aortic wall in patients with an ascending aorta diameter ≥ 5.5 cm, with a lower threshold of 5.0 cm advised in the presence of risk factors [3,4].

Although the exact pathogenesis of TAA is not yet fully understood, several pathological mechanisms such as abnormalities in smooth-muscle cell function and differentiation, influenced by their embryonic origins, have been described [5,6,7]. Clinical risk factors have also been explored in the development of TAAs. While sex-specific variations in TAAs have been clearly documented [8,9], the influence of racial disparities on TAAs remains underexplored, partly due to significant heterogeneity in defining race and ethnicity worldwide. The impact of these disparities on the diagnosis and management of TAAs is crucial for accurate assessment of individual risk levels for a tailored approach. Improved personalized risk stratification is imperative to optimize diagnostic and therapeutic strategies for TAAs among diverse racial groups and to improve patient outcomes. Therefore, this study aims to provide a comprehensive summary of current research on racial disparities in TAAs.

## 2. Materials and Methods

This brief literature review was conducted using a systematic search strategy with the objective of exploring racial differences in the presentation and surgical outcomes of patients with TAAs.

### 2.1. Search Strategy

A comprehensive systematic literature search was performed in PubMed to identify relevant studies published up to 31 December 2023. Controlled search terms were utilized, focusing on two primary domains and encompassing all synonyms of the core terms: “aortic aneurysm” and “race”. In this study, we explicitly focused on TAA. In subsequent screening steps, we further targeted this with specific exclusion criteria to eliminate findings on aortic dissections and abdominal aortic pathology. Additionally, the reference lists of included articles were meticulously cross-checked to ensure no relevant studies were overlooked. For the complete search strategy, see Table 1.

### 2.2. Study Selection

#### 2.2.1. Eligibility Criteria

Inclusion criteria were defined to encompass any empirical study involving patients with TAA that reported on at least two racial groups. Studies were included if they provided predictive values or outcomes such as mortality, readmission rates, or survival rates.

Due to the significant variability in the definition of race or ethnicity and the absence of universally accepted standards for defining ethnic/racial groups, our analyses focused on disparities in terms of race between black and non-black individuals.

Exclusion criteria included review articles, case reports (or studies with a population of fewer than 15), meta-analysis, conference abstracts only published in abstract form, studies published languages other than English, studies on abdominal aneurysms, and studies published prior to 2000.

#### 2.2.2. Screening and Data Extraction

Title and abstract screening were performed by one author (N.B.) to identify potentially relevant articles. Full texts of eligible studies were then assessed according to the predetermined criteria. Data extraction was carried out using a standardized form, which captured information on study population, design, clinical presentation, and surgical outcomes. Separate columns were included for documenting clinical presentation and surgical outcomes for black and non-black patients (Table A1). Data were assessed in a non-quantitative manner.

## 3. Results

### 3.1. Study Selection and Characteristics

**Figure 1:**
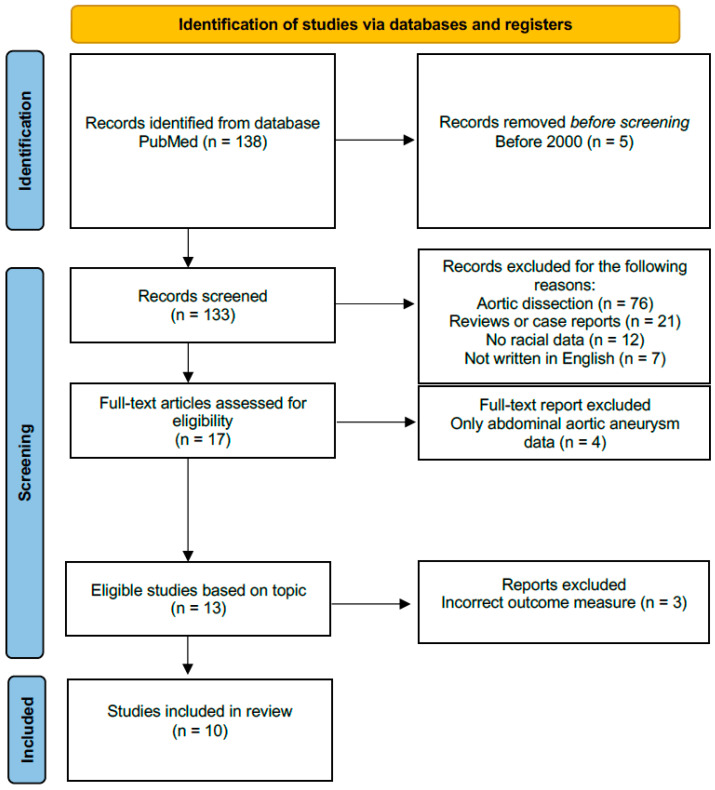
Flowchart of selected articles.

A systematic review of the literature identified 138 unique records. Following title and abstract screening, 121 studies were excluded due to not meeting the inclusion criteria, leaving 17 for full-text review. Of these, we excluded seven studies, of which four focused on abdominal aneurysms and three did not report on relevant outcomes. This resulted in 10 studies being included in the final analysis [10,11,12,13,14,15,16,17,18,19]. See Figure 1 for the flow diagram of this study.

### 3.2. Baseline Characteristics

The included studies collectively reported on 283,076 patients from diverse racial backgrounds, revealing a significant variability in the incidence and clinical presentation of TAA.

Black patients showed a higher incidence of ruptured thoracic aortic aneurysms compared to non-black patients (7.3% vs. 4.4%; *p* = 0.001) [10] among 15,305 patients undergoing TAA repair. Non-black patients tended to present at an older age compared to black patients (74.5 vs. 73.7 years; *p* = 0.001) [10], a finding corroborated by two other studies [13,18].

Black patients also exhibited higher Charlson comorbidity scores (1.51 vs. 0.92; *p* = 0.001) [10], with higher rates of renal insufficiency (35.4% vs. 17.8%; *p* = 0.001) [18], hypertension (100% vs. 86.5%; *p* = 0.034) [18], and diabetes mellitus (18.8% vs. 4.5%; *p* = 0.021) [18], while non-black patients had higher rates of COPD (20.1% vs. 6.3%; *p* = 0.003) [18] and cardiac arrhythmia (20.6% vs. 10.1%; *p* = 0.037) [18]. Two other studies supported these findings [12,17].

Non-black patients had larger aortic necks (28.2 mm vs. 23.8 mm; *p* = 0.01) [17] and a higher prevalence of women with TAA (33.8% vs. 19.3%; *p* = 0.02) [17]. Conversely, two other studies reported a higher prevalence of women among black patients [13,19].

Murphy et al. [15] noted that non-black patients had a higher proportion of elective surgeries (48%; *p* = <0.001), whereas black patients had a higher proportion of emergency surgeries (20%; *p* = <0.001). These findings were confirmed by the study of Yin et al. [11].

### 3.3. Surgical Outcome

Goodney et al. [10] reported on higher perioperative mortality rates during open surgery (14.4% vs. 6.8%; *p* < 0.001) in black patients compared to non-black patients, with black race being a significant risk factor (OR: 2.0; 95% CI 1.5–2.5). However, black patients demonstrated better 5-year survival rates post-open repair (71% vs. 61%; *p* < 0.001).

Overall mortality in black patients was higher (13.7% vs. 9.8%; *p* = <0.001) [15] and significant racial differences in in-hospital mortality were found (*p* < 0.0001) [13]. In line with these results, Ribieras and colleagues [18] reported on higher complication rates (34.3% vs. 17.4%; *p* = 0.014) and conversion rates to open repair (2.9% vs. 0%; *p* = 0.011) in black patients compared to non-black patients.

No significant differences in all-cause or in-hospital mortality between black and non-black patients were found in four studies [12,17,18,19]. Although the study by Yin et al. [11] found no significant cause in overall 30-day mortality rate, after adjustment for demographics, comorbidities, and operative factors, black race was independently associated with a 56% lower 30-day mortality rate after thoracic endovascular aortic repair (TEVAR) (OR: 0.44; 95% CI 0.22–0.85; *p* = 0.01). Johnston et al. [14] also reported varied odds ratios for TEVAR performance across racial groups as outlined in Table A1. Lastly, Vervoort et al. [17] showed a lower reintervention hazard ratio in black patients compared to white patients (HR: 0.7; *p* = 0.01) [17].

## 4. Discussion

This review aimed to summarize current research on racial disparities in the presentation and outcomes of TAAs to aid in the development of personalized risk stratification methods. The analysis of ten empirical studies focusing on the dichotomy between black and non-black individuals revealed that black patients with TAA more commonly exhibit comorbidities such as diabetes, heart failure, and renal insufficiency, whereas non-black patients often present with COPD, coronary artery disease, and cardiac arrhythmias. Black individuals tend to present at a younger age and face a nearly doubled risk of ruptured TAA at presentation compared to non-black individuals. Despite these differences, four studies found no significant disparity between the racial groups [12,17,18,19]. Our findings align with the existing literature on abdominal aortic aneurysms and aortic dissections [20,21,22,23], suggesting potential consistencies in the impact of race across various aortic conditions. Although there are notable disparities in presentation and outcomes of TAA, the underlying mechanisms driving these differences remain unclear. Further research should focus on identifying the underlying factors that contribute to the increased predisposition in certain populations.

The lack of standardization in defining race and ethnicity among studies pose considerable challenges. There is a need for consensus on these definitions to improve the comparability and applicability of research findings. Future research should address these inconsistencies and develop tailored risk assessment models that consider disparities in comorbidities between racial groups. A personalized risk stratification model could enhance the precision in predicting outcomes and improve preventive and therapeutic strategies for patients with TAA. A reasonable suggestion would be to adopt standardized racial categories, such as those defined by the Joint Commitment for Action on Inclusion and Diversity in Publishing [24]. These categories include Asian or Pacific Islander, Black, Hispanic or Latino/a/x, Indigenous (e.g., North American Indian Navajo, South American Indian Quechua, Aboriginal or Torres Strait Islander), Middle Eastern or North African, and White. The classification should prioritize self-reporting by individuals rather than assignment by observers, aligning with recommendations from recent research [25]. This approach would facilitate consistency in defining and analyzing racial disparities in the presentation and outcomes of thoracic aortic aneurysms (TAA), thereby enhancing the validity and comparability of research findings across studies.

While our study compares two groups for comparison—non-black and black patients—it is crucial to recognize that races extend beyond this binary classification. This further underscores the need to advocate for a clear and consistent definition. Within the non-black group, each study comprises a distinct composition of non-black patients which influences our data. Additionally, the scarcity of studies and the fact that those available are mainly from the United States compromise the validity and generalizability of findings. Another noteworthy point is that several studies utilize the same databases, with partially overlapping timeframes. Patient populations may overlap because of this. While this does not pose a significant issue for nonquantitative analysis, it highlights the need for broader, global research to achieve a more comprehensive understanding of these patterns.

In conclusion, this review demonstrates significant differences in the presentation and surgical outcomes of TAA between racial groups. Recognizing these differences is essential for developing tailored interventions and improving outcomes for all patients, regardless of race or ethnicity. Further research is needed to uncover the underlying cause of these disparities and to refine risk stratification models accordingly.

## Figures and Tables

**Table 1:** Search strategy: PubMed.

Search	PubMed Queries—31 December 2023	Results
#1		17,667
	“Aortic Aneurysm, Thoracic” [MeSH Terms] OR “Thoracic Aortic Aneurysm *” [Title/Abstract] OR “Aneurysm, Thoracic Aortic” [Title/Abstract] OR “Aneurysm, Thoracic Aorta” [Title/Abstract] OR “Aorta Aneurysm *, Thoracic” [Title/Abstract] OR “Thoracic Aorta Aneurysm *” [Title/Abstract] OR “Thoracic Aortic Aneurysms” [Title/Abstract] OR “Thoracic Aortic Aneurysm *” [Title/Abstract] OR “Thoracic aneurysm *” [Title/Abstract]	
#2		761,284
#3	“ethnology” [MeSH Terms] OR “ethnicity” [MeSH Terms] OR “ethnic group *” [Title/Abstract] OR “population groups” [MeSH Terms] OR “racial groups” [MeSH Terms] OR “racial groups” [Title/Abstract] OR “ethnicity” [Title/Abstract] OR “race” [Title/Abstract] OR “Epidemiology” [MeSH Terms] OR “Epidemiology” [Title/Abstract] OR “Race-based” [Title/Abstract] OR “Inter-ethnic” [Title/Abstract]	138
	((“aortic aneurysm, thoracic ” [MeSH Terms] OR “thoracic aortic aneurysm *” [Title/Abstract] OR “aneurysm thoracic aortic” [Title/Abstract] OR “aneurysm thoracic aorta” [Title/Abstract] OR “aorta aneurysm * thoracic” [Title/Abstract] OR “thoracic aorta aneurysm *” [Title/Abstract] OR “Thoracic Aortic Aneurysms” [Title/Abstract] OR “thoracic aortic aneurysm *” [Title/Abstract] OR “thoracic aneurysm *” [Title/Abstract]) AND (“ethnology” [MeSH Terms] OR “ethnicity” [MeSH Terms] OR “ethnic group *” [Title/Abstract] OR “population groups” [MeSH Terms] OR “racial groups” [MeSH Terms] OR “racial groups” [Title/Abstract] OR “ethnicity” [Title/Abstract] OR “race” [Title/Abstract] OR “Epidemiology” [MeSH Terms] OR “Epidemiology” [Title/Abstract] OR “Race-based” [Title/Abstract] OR “Inter-ethnic” [Title/Abstract]))	
Overall		138

## Data Availability

No new data were created or analyzed in this study. Data sharing is not applicable.

## Appendix A

**Table A1:** Description of the ten included articles and their characteristics.

First Author (Year)	Study Type (Method)	Study Population(s) (Controls and Patients)	Clinical Presentation		Surgical Outcome	
			Non-Black	Black	Non-Black	Black
Goodney (2013) [10]	Retrospective cohort study Intervention: Thoracic aneurysm repair Control: Mortality Data source: Medicare claims (1999–2007)	*n* = 722 black patients *n* = 14,583 non-black patients (97% white, 1.0% Native American, 0.9% Hispanic, 0.9% Asian American, 0.1% Pacific Islander, 0.1% missing)	Older presentation (74.5 vs. 73.7; *p* = 0.001); 4,4% ruptured TAA (7.3% vs. 4.4%; *p* = 0.001); non-black patients had a higher ratio of men (56.4% vs. 43.4%; *p* = 0.02)	Younger presentation (74.5 vs. 73.7; *p* = 0.001) 7.3% ruptured TAA (7.3% vs. 4.4%; *p* = 0.001) Black patients had a higher Charlson comorbidity score (1.51 vs. 0.92; *p* = 0.001). Black patients had a higher prevalence of diabetes, heart failure, renal failure and history of malignancy (*p* = 0.001).	Open surgical repair: lower perioperative mortality 6.8% non-black; *p* < 0.001; 5-year survival: 61% *p* < 0.001	Open surgical repair: higher perioperative mortality 14.4% black; *p* < 0.001. Operative mortality: OR 2.0; 95% CI 1.5–2.5; *p* < 0.0001. 5-year survival: 71%; *p* < 0.001.
Yin (2021) [11]	Retrospective cohort study Intervention: Thoracic endovascular aneurysm repair Control: 30-day mortality Data source: VQI national data registry	*n* = 684 black patients *n* = 2021 non-black patients (100% white)	1488 aneurysms (73.6%)	More likely to undergo emergent TEVAR (27.6% vs. 19.8%; *p* < 0.001). More likely symptomatic (52.3% vs. 36.4%; *p* < 0.001). More likely to receive blood transfusion (32.1% vs. 23.6%; *p* < 0.001).	30-Day Mortality: No significant difference in 30-day mortality: (3.4% vs. 4.9%; *p* = 0.1)	30-Day Mortality: Following correction for operative variables, comorbidities, and demographics, black race was independently associated with 56% decrease in risk after Tevar (OR 0.44; 95% CI 0.22–0.85; *p* = 0.01). Postoperative Complications: No independent association (OR 0.90; 95% CI 0.68–1.17; *p* = 0.42). 1-year overall survival: log-rank *p* = 0.024. 1-year mortality Hr:0.65; 95% CI, 0.47–0.91; *p* = 0.01.
Diaz-Castrillon (2022) [12]	Retrospective cohort study Intervention: Thoracic endovascular aneurysm repair Control: In-hospital mortality Data source: Nationwide inpatient sample (NIS) 2010–2017	*n* = 4959 black *n* = 20,301 non-black (68.1% white, 5.7% Hispanic, 6.5% others)	CAD more prevalent (34.6% (white) vs. 24.1% (black) vs. 26.8% (Hispanic) vs. 24.7% (others); *p* < 0.001). COPD more prevalent (28.7% vs. 15.6% vs. 15.1% vs. 16.5%; *p* < 0.001). TEVAR often times elective (58.8% vs. 34% vs. 48.3% vs. 48.2%; *p* < 0.001)	Hypertension more frequent as a comorbidity (92% (black) vs. 83% (white) vs. 85% (Hispanic) vs. 84% (others); *p* < 0.001).	Racial disparities do not appear to be associated with in-hospital mortality	Racial disparities do not appear to be associated with in-hospital mortality.
Tanious (2019) [13]	Retrospective cohort study Intervention: Thoracic endovascular aneurysm repair Control: In-hospital mortality Data source: Florida State Agency for Health Care Administration 2000–2014	*n* = 1630 black *n* = 34,119 non-black (47.7% white, 46.0% Hispanic, 1.8% other)	Older presentation 67.42 (black) vs. 73.87 (white) vs. 73.52 (Hispanic) vs. 72.06 (other); *p* < 0.001	Higher prevalence of women 31.5% (black) vs. 16.1 (white) vs. 20.2 (Hispanic) vs. 21.8 (other); *p* < 0.001.	Chance of in-hospital mortality: 2.5% (white), 2.8% (Hispanic), 5.1% (other); *p* < 0.0001	Chance of in-hospital mortality: 4.0%; *p* < 0.0001
Johnston (2013) [14]	Retrospective cohort study Intervention: Thoracic endovascular aneurysm repair Control: TEVAR performance based on race Data source: Nationwide inpatient sample (NIS) 2005–2008	*n* = 4108 black *n* = 41,122 non-black (86% white, 6.2% Hispanic, 3.2% Asian or Pacific Islander, 0.8% Native American, 3.7% other)	NA	A total of 28.6% of black patients received TEVAR, whereas only 19.5% of white patients were treated with TEVAR (*p* < 0.001).	TEVAR performance: Odds ratio: Native American: 2.37 Black: 1.71 Hispanic: 1.70 Asian or Pacific Islander: 1.34 Other: 0.98 White (reference): 1	Tevar performance: Odds: Black: 1.71
Murphy (2013) [15]	Retrospective cohort study Intervention: Thoracic endovascular aneurysm repair Control: mortality Data source: Nationwide inpatient sample (NIS) 2001–2005	*n* = 819 black *n* = 9738 non-black (88% white, 5.7% Hispanic, 6.8 other)	High prevalence of elective surgery: 48%; *p* < 0.001	High prevalence of emergency surgery: 20%; *p* < 0.001.	Mortality rate: 9.8%; *p* < 0.001	Mortality rate: 13.7%; *p* < 0.001
Abdulameer (2019) [16]	Retrospective cohort study Intervention: Thoracic aneurysm rupture Control: Mortality per million Data source: U.S. National Vital Statistics System 1999–2016	*n* = 104,458 total ruptures	NA	NA	Mortality/million: White women: 3.5; White men: 3.3; Asian men: 1.5; Asian women: 2.5 (*p* < 0.001)	Mortality/million Black women: 2.3 Black men: 2.6 (*p* < 0.001)
Vervoort (2021) [17]	Retrospective cohort study Intervention: Elective thoracic endovascular aneurysm repair Control: Reintervention and surgical outcome Data source: Vascular Quality Initiative 2009–2018	*n* = 2140 black *n* = 40,431 Non-black (100% white)	Women sex 33.8% (23), *p* = 0.02; Aortic neck in mm 28.2 +/−15.8 *p* = 0.01; CHF: 6.0 (4) *p* = 0.01; Smoking history: 89.7 (61) *p* < 0.01	Female sex 19.3% (212), *p* = 0.02; Aortic neck in mm 23.8+/−5.25 *p* = 0.01; CHF: 13.0 (143) *p* = 0.01; Smoking history: 83.1 (911) *p* < 0.01.	All-cause mortality: similar between groups (log-rank *p* = 0.25); Reintervention: white race statistically associated with reintervention; *p* = 0.01	All-cause mortality: similar between groups (log-rank *p* = 0.25) Reintervention: Hr: 0.7; *p* = 0.01
Ribieras (2023) [18]	Retrospective cohort study Intervention: thoracic endovascular aneurysm repair Control: All-cause mortality Data source: Global Registry for Endovascular Aortic Treatment (GREAT) 2010–2016	*n* = 79 black *n* = 359 non-black	Chronic obstructive pulmonary disease: black 6.3% vs. white 20.1%; *p* = 0.003; Cardiac arrhythmia: black 10.1% vs. white 20.6%; *p* = 0.037	Younger presentation: 62 years vs. 67 years); *p* < 0.001. Higher BMI 31.0 kg/m^2^ vs. 27.5 kg/m^2^); *p* < 0.001. Renal insufficiency: 35.4% vs. 17.8%; *p* = 0.001. Higher incidence of erectile dysfunction in black patients 6.3% vs. 2.0%; *p* = 0.047. Higher incidence of hypertension: common in black patients (100% vs. 86.5%; *p* = 0.034). Higher prevalence of diabetes mellitus: 18.8% vs. 4.5%; *p* = 0.021.	All-cause mortality: no significant difference	Complications: 34.3% vs. 17.4%; *p* = 0.014 Conversion to open repair: 2.9% vs. 0%; *p* = 0.011 Type II endoleaks: 5.7% vs. 1.0%; *p* = 0.040 All-cause mortality: no significant difference
Murphy (2010) [19]	Retrospective cohort study Intervention: Thoracic aneurysm rupture Control: Mortality Data source: U.S. National Vital Statistics System 2001–2005	*n* = 104 black *n* = 699 non-black (93% white, 7% Hispanic)	Men: 450/650 (white), 32/49 (Hispanic); *p* < 0.001	Men: 54/104 *p* < 0.001.	Overall mortality: 13.3% (*n* = 117), no differences between patients of varied ethnicity; Mortality: 12% (white), 10% (Hispanic), 19% (other); *p* = 303	Overall mortality: 13.3% (*n* = 117), no differences between patients of varied ethnicity Mortality: 12% died; *p* = 0.303

NA—not applicable.
